# Editorial for the Special Issue “Anticancer Activity and Metabolic Pathways of Natural Products 2.0”

**DOI:** 10.3390/biomedicines13092083

**Published:** 2025-08-27

**Authors:** Seung Ho Baek

**Affiliations:** College of Korean Medicine, Dongguk University, 32 Dongguk-ro, Ilsandong-gu, Goyang-si 10326, Republic of Korea; baekone99@gmail.com

## 1. Editorial Summary

Cancer progression involves hallmark features, including sustained proliferative signaling, evasion of apoptosis, metastatic spread, and metabolic reprogramming. Therapeutic strategies increasingly target these diverse pathways to curb tumor growth and resistance mechanisms. Natural products have attracted considerable interest due to their multifaceted actions on cancer cells and the tumor microenvironment, as well as generally favorable safety profiles. Their versatility in modulating signaling, inducing cell death, and alleviating toxicity positions them favorably within emerging precision oncology paradigms [1,2,3,4,5,6].

## 2. Anticancer Potential of Steroidal Saponins

Steroidal saponins from Paris polyphylla demonstrate the capacity to inhibit colorectal cancer cell growth by orchestrating apoptosis and disrupting cancer metabolism. These multi-target effects highlight the potential of natural compounds as adjuncts or alternatives in chemotherapeutic regimens.

## 3. Protective and Adjuvant Roles of Cytokinin-Related Molecules

Certain cytokinins show promise in reducing chemotherapy-induced toxicity through antioxidative and anti-inflammatory mechanisms, thus potentially improving patient tolerability and therapeutic indices.

## 4. Targeting Transcription Factors with Small Molecule Inhibitors

Targeting oncogenic transcription factors such as FoxM1 with selective inhibitors has emerged as an effective strategy to suppress tumor cell proliferation and induce apoptosis. These approaches leverage deeper insights into cancer gene regulation acquired through advances in molecular biology.

## 5. Flavonoids: Multifunctional Modulators of Cancer Signaling and Metabolism

Polyphenolic flavonoids, widely distributed in fruits and vegetables, regulate multiple signaling pathways including NF-κB, PI3K/Akt/mTOR, and Wnt. They can promote apoptosis, cell cycle arrest, inhibit metastasis, and modulate reactive oxygen species (ROS) with dual roles in cancer prevention and treatment [7].

## 6. Dual Induction of Apoptosis and Ferroptosis by Alkaloids

Some natural alkaloids induce cell death by simultaneously activating apoptosis and ferroptosis, a lethal iron-dependent oxidative process. This multi-modal death induction offers a promising approach to overcoming resistance in various malignancies.

## 7. Emerging Roles of Triterpenoids and Polysaccharide-Peptides

Triterpenoids and polysaccharide-peptides derived from plants and fungi act on nuclear receptors and kinases, with demonstrated anti-inflammatory and anticancer effects. Their low toxicity profile facilitates research into combination therapies and novel immunomodulatory cancer treatments [8].

## 8. Nanoparticles and Thermal Enhancement in Cancer Treatment

Nanotechnology integrated with hyperthermia enhances selective tumor ablation through ROS generation and heat shock protein modulation. Hyperthermia synergizes with natural compounds, improving apoptosis induction and impairing stress response pathways in cancer cells [9,10,11,12].

## 9. Emergent Biomarkers and Therapeutic Targets

Hyaluronic acid (HA) and its receptor CD44 influence tumor progression, metastasis, and drug resistance, especially in female cancers. These molecules represent promising biomarkers and therapeutic targets for personalized treatment strategies [13].

## 10. Glutathione Metabolism and Implications for Cancer Therapy

Glutathione (GSH) plays a pivotal role in maintaining redox homeostasis; its dysregulation contributes to chemotherapy resistance. Therapeutic modulation of GSH pathways holds potential but requires balancing to protect normal cells while targeting tumor cells effectively.

## 11. Small Molecule Steroid Derivatives with Epoxy Functionality

Epoxy (oxirane)-containing natural steroids exhibit anticancer activities, including kinase inhibition and apoptosis induction. Exploration of their structure-activity relationships primes development of multi-target therapeutics suitable for complex cancer phenotypes.

## 12. Conclusions and Perspectives

These compiled insights emphasize the multifaceted potential of natural products in cancer therapy—simultaneously targeting multiple hallmarks of cancer and improving conventional treatments. Addressing challenges such as bioavailability and clinical validation remains necessary for successful translation. Integrative approaches combining chemistry, molecular biology, nanotechnology, and physical modalities like hyperthermia are indispensable for advancing natural product-based precision oncology.
